# 0434. Simulation of late inspiratory rise in airway pressure during pressure support ventilation

**DOI:** 10.1186/2197-425X-2-S1-P26

**Published:** 2014-09-26

**Authors:** C-H Yu, P-L Su, W-C Lin, C-W Chen

**Affiliations:** National Cheng Kung University Hospital Dou-Liou Branch, Department of Internal Medicine, Yun-Lin, Taiwan Province of China; National Cheng Kung University Hospital, Department of Internal Medicine, Tainan, Taiwan Province of China

## Introduction

**Figure 1:**
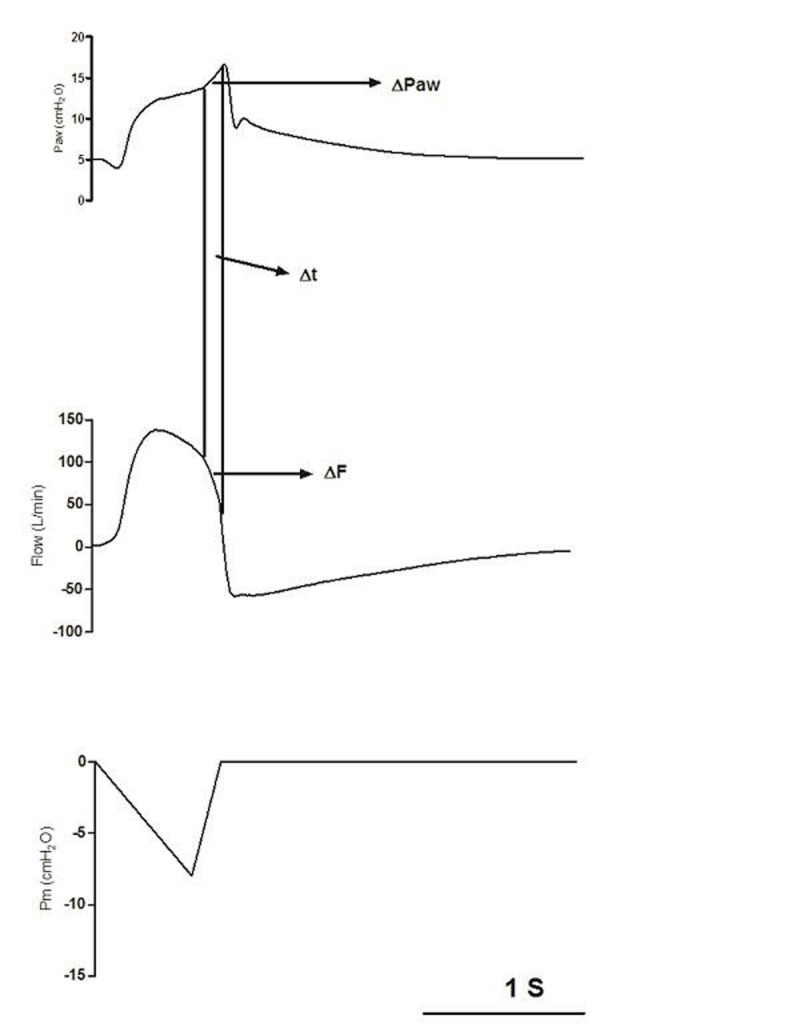
Figure 1

Late inspiratory rise in airway pressure (LIRAP, ΔPaw/Δt, Fig. 1) caused by inspiratory muscle relaxation or expiratory muscle contraction is frequently seen during pressure support ventilation [1, 2], although the factors that modulate LIRAP are unknown.

## Objectives

The aim of current study was to determine the factors related to LIRAP during PSV using a simulation lung model. Influence of inspiratory muscle relaxation was assessed under various combinations of lung models and ventilator settings. Influence of expiratory muscle contraction was assessed through its intervention at various time points during the inspiratory phase.

## Methods

We investigated the effects of respiratory mechanics (normal, obstructive, restrictive, or mixed), inspiratory effort (-2, -8, or -15 cmH_2_O), flow cycling-off criteria (5-40% peak inspiratory flow), and duration of inspiratory muscle relaxation (0.18-0.3 s) on LIRAP during pressure support ventilation using a lung simulator (ASL 5000) and four types of ventilators. A user-defined breath was used to simulate expiratory muscle contraction. Seven breaths were recorded for each scenario.

## Results

LIRAP occurred within all lung models when inspiratory effort was medium to high and duration of inspiratory muscle relaxation was short. The normal lung model was associated with the fastest LIRAP (maximum ranged from 24.8 to 46.1 cmH_2_O/s in four types of ventilators), whereas the obstructive lung model was associated with the slowest LIRAP (maximum from 11.1 to 25.1 cmH_2_O/s, p < 0.0001 between different ventilators and different lung models). Unless lung mechanics were normal, LIRAP was unlikely to occur when inspiratory effort was low. Different ventilators were also associated with differences in LIRAP speed (p < 0.0001). Lowest ΔPaw/Δt was recorded in PB-840 ventilator and highest ΔPaw/Δt in GE Carestation ventilator in all lung models. Except for within the restrictive lung model, changes in flow-cycling level did not abolish LIRAP if inspiratory effort was medium to high. Increased duration of inspiratory relaxation also led to the elimination of LIRAP. Simulation of expiratory muscle contraction revealed that LIRAP occurred only when expiratory muscle contraction occurred sometime after the beginning of inspiration.

## Conclusions

Our simulation study reveals that both respiratory resistance and compliance may affect LIRAP. Except under restrictive lung conditions, LIRAP is unlikely to be abolished by simply lowering flow-cycling criteria when inspiratory effort is strong and relaxation time is rapid.
